# Neoadjuvant nivolumab plus chemotherapy followed by resection for superior sulcus tumour with high PD‐L1 expression: A case report

**DOI:** 10.1002/rcr2.1358

**Published:** 2024-04-25

**Authors:** Takahito Nakaya, Yoshimitsu Hirai, Hiroaki Akamatsu, Fumiyoshi Kojima, Hideto Iguchi, Aya Fusamoto, Yumi Yata, Takahiro Nagai, Daiki Kitahara, Toshiaki Takakura, Yoshiharu Nishimura, Nobuyuki Yamamoto

**Affiliations:** ^1^ Department of Thoracic and Cardiovascular Surgery Wakayama Medical University Wakayama Japan; ^2^ Internal Medicine III Wakayama Medical University Wakayama Japan; ^3^ Department of Human Pathology Wakayama Medical University Wakayama Japan

**Keywords:** minimally invasive surgery, neoadjuvant chemotherapy, nivolumab, non‐small cell lung cancer, superior sulcus tumour

## Abstract

The standard treatment for resectable non‐small cell lung cancer (NSCLC) located in the superior sulcus is neoadjuvant chemoradiotherapy followed by highly invasive resection. Based on the results of the CheckMate 816 trial, which showed a marked improvement in the efficacy of neoadjuvant chemo‐immunotherapy, we report a case of minimally invasive resection after neoadjuvant nivolumab plus chemotherapy for superior sulcus NSCLC, resulting in a pathologic complete response. The patient was a 76‐year‐old man with a 65‐mm right superior sulcus tumour diagnosed as squamous cell carcinoma with 95% PD‐L1. After two courses of neoadjuvant nivolumab plus chemotherapy, the tumour was completely resected through an 11‐cm right lateral thoracotomy with second rib resection and first rib preservation. No residual tumour cells were observed in the specimen, and the patient had a pathologic complete response. This report represents a new treatment option for superior sulcus tumours.

## Graphical abstract

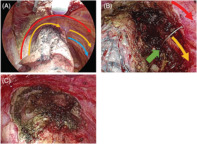

## INTRODUCTION

Neoadjuvant chemoradiotherapy, followed by resection, is recommended for patients with non‐small cell lung cancer (NSCLC) involving the chest wall located in the superior sulcus.^1^ However, the typical surgical procedure for superior sulcus tumour (SST) with dorsal superior rib resection is highly invasive. The CheckMate 816 trial revealed that neoadjuvant nivolumab plus chemotherapy is a new treatment strategy for resectable NSCLC,^2^ but its efficacy in SST is unclear. Here, we report a case of SST with high PD‐L1 expression in a patient who underwent complete resection via minimally invasive surgery after receiving neoadjuvant nivolumab plus chemotherapy.

## CASE REPORT

**FIGURE 1:**
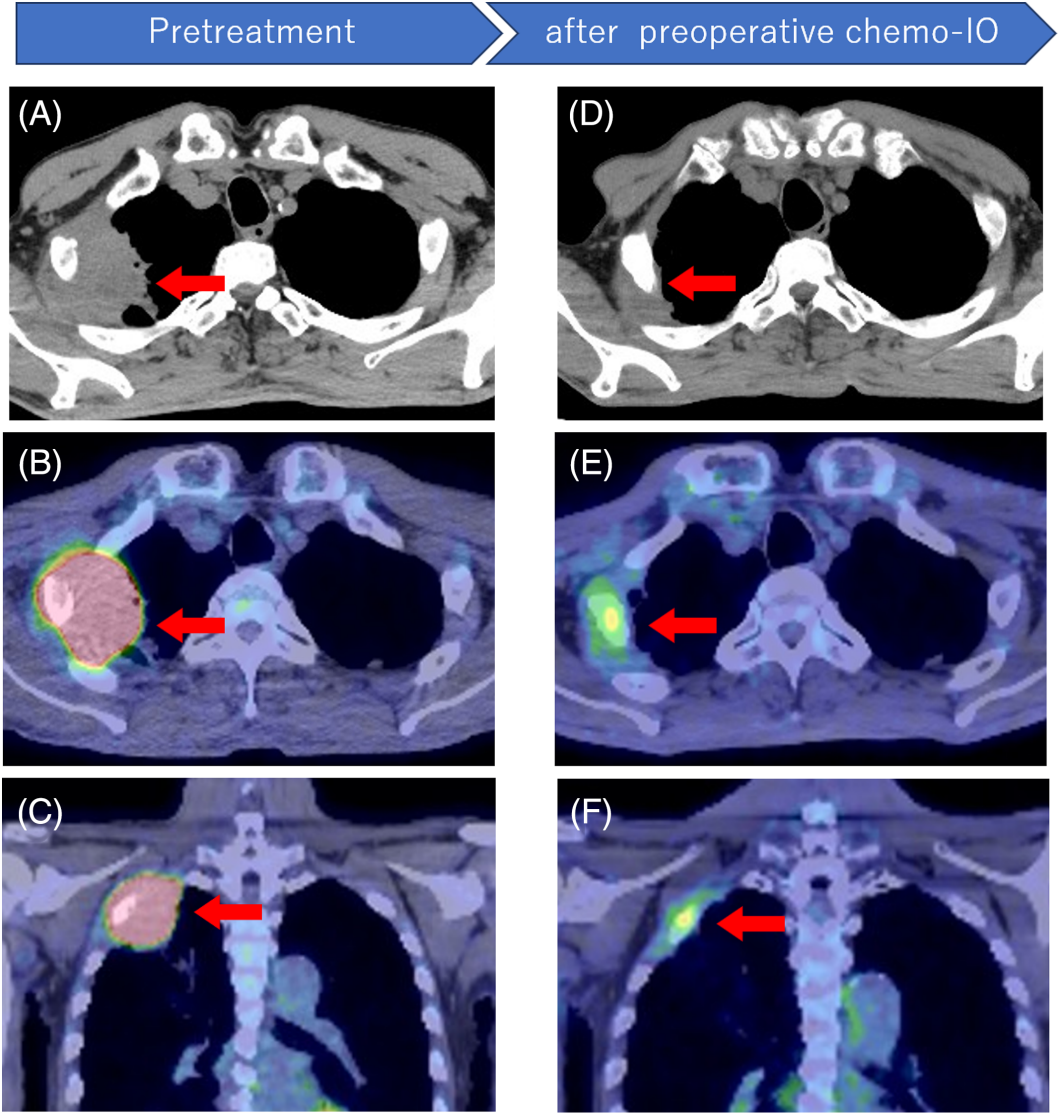
Computed tomography (CT) at first visit to our hospital in axial view (A), Positron emission tomography‐computed tomography (PET‐CT) at first visit to our hospital in axial (B) and coronal view (C), CT after neoadjuvant nivolumab and chemotherapy in axial view (D), PET‐CT after neoadjuvant chemo‐immunotherapy in axial (E) and coronal view (F).

A 76‐year‐old man with right‐sided chest pain was referred to our hospital. Computed tomography (CT) showed a 65‐mm tumour located in the apical pulmonary region, which invaded the chest wall and showed osteolytic changes in the second rib (Figure 1A). Positron emission tomography‐computed tomography (PET‐CT) revealed a high maximum standardized uptake of fluorodeoxyglucose (FDG) in the tumour and no other affected lymph nodes or organs on whole‐body imaging (Figure 1B,C). After transbronchial lung biopsy, the tumour was diagnosed as squamous cell carcinoma of the lung with PD‐L1 expression of 95% by immunohistochemistry (22c3, Dako). Neoadjuvant chemotherapy (carboplatin [AUC 6], paclitaxel [200 mg/m^2^], and nivolumab [200 mg/body]) was started and after two courses of treatment, the tumour size shrank from 65 mm to 21 mm (Figure 1D) and areduction of FDG uptake was observed reduction of maximum standardized FDG uptake was observed on PET‐CT (18.8–3.71, Figure 1E,F). Despite good local control, the patient's general condition deteriorated slightly. Therefore, we decided to perform a resection.

**FIGURE 2:**
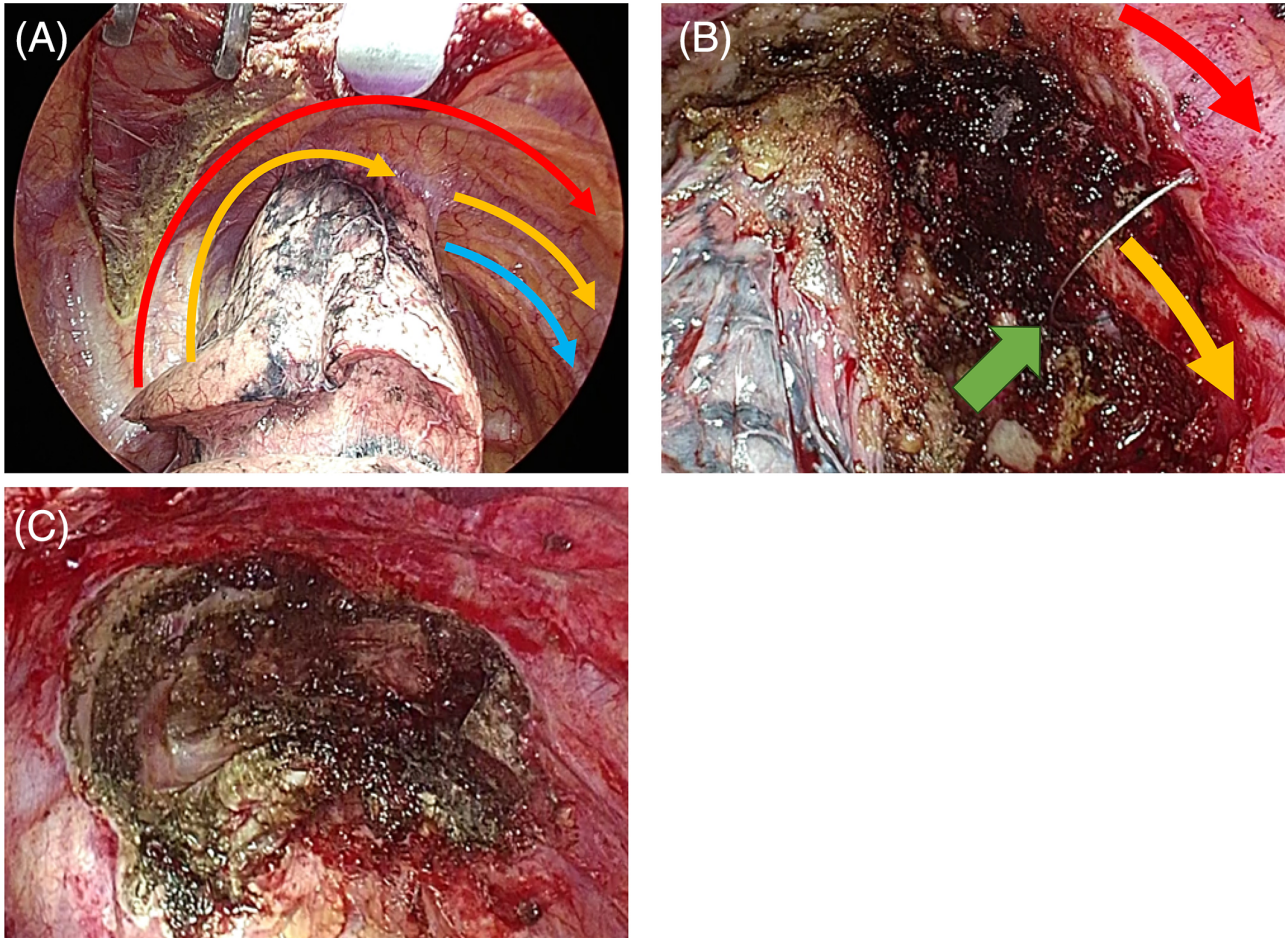
Intraoperative view. Arrows show the location of ribs. Blue arrow: first rib, yellow arrow: second rib, red arrow: third rib, green arrow: line saw.

The patient was placed in the left lateral position under general anaesthesia using a double‐lumen endotracheal tube. The surgical procedure was performed through an 11‐cm right lateral thoracotomy at the fourth intercostal space between the middle and posterior axillary lines. Because the lung adhesions were limited to tumour invasion, it was determined that resection of the first rib was not necessary and no additional open chest surgery was performed (Figure 2A). The second rib was resected dorsally using Riehl and Kellison forceps and ventrally using a line saw guided by an indwelling needle sheath (Figure 2B). The periostea of the first and third ribs were dissected in the extrapleural layer along with the resected second rib, and the portion of the chest wall invaded with tumour was resected from within the chest cavity. Subsequently, with the resected chest wall attached to the lung, a right upper lobectomy and mediastinal lymph node dissection were performed, and the tumour was removed en bloc (Figure 2C). The operative time was 316 min and blood loss was 165 mL. Postoperative pain meditation was as usual, effective and well pain control was obtained. The drainage tube was removed on the third postoperative day, and the patient was discharged on the eighth day without postoperative complications. Histopathological findings showed no residual tumour, and the patient was diagnosed with pathologic complete response (pCR; residual viable tumour: 0%). Surgical margins were negative. After three postoperative months, the patient is alive without postoperative complications, and remained in good general condition.

## DISCUSSION

Because local control is difficult with resection alone, neoadjuvant chemoradiotherapy followed by resection is the standard treatment for SST.^1^ Even if chemoradiotherapy causes substantial tumour shrinkage, it often provokes inflammation and fibrosis around the tumour, making resection difficult. Rusch et al. reported the results of neoadjuvant chemoradiotherapy in patients with SST in the Intergroup Trial 0160 study,^3^ with a pCR rate of 36%. In contrast, the CheckMate 816 trial showed a comparable pCR rate, even though this regimen did not include radiotherapy. The pCR rate of nivolumab plus chemotherapy was 44.7% in patients in whom PD‐L1 was expressed in more than 50% of the tumour.^1^

In this case, the tumour showed 32% radiological shrinkage, and more importantly, we successfully resected the tumour using a less invasive method. Because radiotherapy was omitted, estimating the extent of tumour invasion during surgery without adhesions or fibrosis caused by radiotherapy proved beneficial. Resection for SST with dorsal high‐rib resection usually requires a large skin incision and extensive muscle resection, such as high posterolateral thoracotomy,^4^ which results in postoperative pain and a limited range of motion of the upper extremities. In this case, we prepared with high posterolateral thoracotomy and minimally invasive approach. As expected, the excellent intraoperative visual field enabled second rib resection to be performed within the thoracic cavity using a minimally invasive approach, and pCR was achieved (residual viable tumour: 0%). Although observation of recurrence required long‐term follow‐up, the patient had no postoperative complications such as postoperative pain and a limited range of motion of the upper extremities. This treatment strategy may have resulted in a good postoperative course.

## AUTHOR CONTRIBUTIONS

**Takahito Nakaya**: Conceptualization; writing‐original draft and visualization. **Yoshimitsu Hirai**: Supervision and writing‐original draft and review and editing and visualization. **Hiroaki Akamatsu, Fumiyoshi Kojima, Hideto Iguchi, Aya Fusamoto, Yumi Yata, Takahiro Nagai, Daiki Kitahara, Toshiaki Takakura, Yoshiharu Nishimura, Nobuyuki Yamamoto**: Project administration and writing‐review and editing.

## CONFLICT OF INTEREST STATEMENT

Hiroaki Akamatsu, Honoraria; Amgen Inc, AstraZeneca K.K., Boehringer Ingelheim Japan Inc., Bristol‐Myers Squibb, Chugai Pharmaceutical Co. Ltd., Eli Lilly Japan K.K., MSD K.K., Nippon Kayaku. Co. Ltd., Novartis Pharma K.K., Ono Pharmaceutical Co. Ltd., Pfizer Inc, Takeda Pharmaceutical Co. Ltd. and Taiho Pharmaceutical Co. Ltd. Advisory role; Amgen Inc, and Janssen Pharmaceutical K.K., Sandoz. Research funding; Amgen Inc, Chugai Pharmaceutical Co. Ltd. and MSD K.K.. Nobuyuki Yamamoto, Honoraria; AbbVie Inc., Accuray Japan K.K., Amgen K.K., AstraZeneca K.K., Boehringer Ingelheim Japan Inc., Chugai Pharmaceutical Co. Ltd., Daiichi Sankyo Co., Ltd., Eli Lilly Japan K.K., Guardant Health Japan Corp., Janssen Pharmaceutical K.K., Merck Biopharma Co., Ltd., Miyarisan Pharmaceutical Co., Ltd., MSD K.K., Kyorin Pharmaceutical Co., Ltd., Novartis Pharma K.K., Ono Pharmaceutical Co. Ltd., Pfizer Inc, Takeda Pharmaceutical Co. Ltd. and Taiho Pharmaceutical Co. Ltd., Terumo Corp., Tsumura & Co.. Advisory role; Amgen K.K., AstraZeneca K.K., Chugai Pharmaceutical Co. Ltd., Eli Lilly Japan K.K., MSD K.K., Novartis Pharma K.K., Ono Pharmaceutical Co. Ltd. Research funding; AbbVie Inc., Amgen K.K., Asahi Kasei Corporation, AstraZeneca K.K., A2 Healthcare Corporation, Boehringer Ingelheim Japan Inc., Bristol‐Myers Squibb K.K., Chugai Pharmaceutical Co. Ltd., EPS Holdings, Inc., IQVIA Services Japan K.K., Janssen Pharmaceutical K.K., Mebix, Inc., MSD K.K., Novartis Pharma K.K., Ono Pharmaceutical Co. Ltd., Taiho Pharmaceutical Co. Ltd.. Data safety Monitoring Board: AstraZeneca K.K. All other authors declare no conflict of interest.

## ETHICS STATEMENT

The authors declare that appropriate written informed consent was obtained for the publication of this manuscript and accompanying images.

## Data Availability

The data that support the findings of this study are available on request from the corresponding author, Yoshimitsu Hirai.
